# The Prognostic Correlation of Heart Rate Variability at Diagnosis with Survival of Patients with Hepatocellular Carcinoma

**DOI:** 10.3390/diagnostics11050890

**Published:** 2021-05-17

**Authors:** Ana-Maria Ciurea, Dan Ionuț Gheonea, Michael Schenker, Alina Maria Mehedințeanu, Georgică Costinel Târtea, Cristin Constantin Vere

**Affiliations:** 1 Department of Oncology, University of Medicine and Pharmacy of Craiova, 200349 Craiova, Romania; amciurea14@gmail.com (A.-M.C.); michael.schenker@umfcv.ro (M.S.); alina.maria591@gmail.com (A.M.M.); 2 Department of Gastroenterology, University of Medicine and Pharmacy of Craiova, 200349 Craiova, Romania; cristin.vere@umfcv.ro; 3 Department of Physiology, University of Medicine and Pharmacy of Craiova, 200349 Craiova, Romania; 4 Department of Cardiology, Emergency County Hospital of Craiova, 200642 Craiova, Romania

**Keywords:** hepatocellular carcinoma, heart rate variability, biomarker prognosis

## Abstract

Background: Heart rate variability (HRV) indices have been shown to be associated with prognosis in various types of cancer. This study aims to assess the ability of these indices to predict survival in hepatocellular carcinoma (HCC) patients after diagnosis. Methods: We retrospectively collected data from 231 patients diagnosed with HCC between January 2014 and March 2018. The baseline clinical-pathological variables and HRV indices (extracted from Holter electrocardiogram recordings) were analyzed. Results: Univariate and multivariate analyses were performed to identify the predictive value of the above factors for overall survival (OS). The univariate analysis revealed that an age > 60 years, hepatitis C, portal vein involvement (thrombosis), a tumor size > 5 cm, alpha-fetoprotein (AFP) > 400 ng/mL, serum albumin, and C-reactive protein (CRP) were risk factors for poor OS. Multivariable Cox regression analyses identified that a tumor size > 5 cm and AFP > 400 ng/mL predict poorer outcomes in HCC patients. It should be mentioned that, in both the univariate analysis and in the multivariate analysis, between HRV indices, SDNN (standard deviation of all normal-to-normal (NN) intervals) < 110 ms was an independent risk factor for OS with an HR of 3.646 (95% CI 2.143 to 6.205). Conclusion: This study demonstrates that HRV indices identify HCC patients at high risk of death and suggests that such monitoring might guide the need for early therapy in these types of patients, as well as the fact that HRV can be a potential noninvasive biomarker for HCC prognosis.

## 1. Introduction

Hepatocellular carcinoma (HCC) was the most common type of liver cancer recorded worldwide in 2020, affecting both genders and all ages, with 905,677 new cases (5%), ranking sixth after breast, lung, prostate, colon, and stomach cancer [1,2]. If the incidence is 5% worldwide, mortality for this type of cancer occupies the third position (after lung and colorectum), and in 2020, there were approximately 830,180 deaths, accounting for approximately 8.3% of all cancer deaths [1,2]. Since the 5-year survival rate for this primary malignancy of the liver is 18%, HHC has thus become the second most lethal type of tumor after pancreatic cancer [3,4]. Most cases of HCC occur in patients who have liver disease, who consume excessive alcohol, who are infected with hepatitis B or C virus, or, as in Western countries, in patients with nonalcoholic fatty liver disease (NAFLD), metabolic syndrome, or obesity [5]. A diagnosis of HHC is established primarily by imaging explorations (ultrasonography (US), computed tomography, magnetic resonance imaging (MRI), and angiography) and via serum biomarkers (the most used biomarker being Alpha-fetoprotein (AFP)) [5,6,7]. Therefore, not only to obtain a good prognosis but also to improve clinical outcomes, the identification of new and reliable noninvasive biomarkers, or a combination of markers, is of paramount importance.

Heart rate variability (HRV) is the change in time intervals between consecutive heartbeats [8]. In the case of a healthy heart, they are complex and constantly changing. This allows the cardiovascular system to adapt quickly to sudden physical and psychological changes in order to maintain homeostasis [8]. In terms of the clinical relevance of HRV, it was first discussed in 1965 by Hon and Lee, who identified that fetal distress was preceded by changes in inter-beat intervals before the heart rate changed significantly [9]. Twenty years ago, Sayers and others described the fact that physiological rhythms are part of the beat-to-beat heart rate signal [10]. In recent years, the assessment of heart rate variability (HRV) has become an easily applicable and reliable tool in clinical practice for the analysis of sympathetic and parasympathetic influences in patients with neurological and psychiatric disorders [11,12], cardiovascular disorders [13,14], or cancer [15,16,17]. In cancer, several studies indicate that a reduction in HRV is common in these patients, with this observation signifying the existence of an autonomous dysfunction that is associated with the disease [18]. In addition, several studies have reported a correlation between HRV indices and the overall progression and survival of cancer patients. An increase in HRV indices may be associated with a better prognosis in patients suffering from different types of cancer [18].

This study aims to assess the ability of HRV indices to predict survival in hepatocellular carcinoma (HCC) patients after diagnosis.

## 2. Materials and Methods

This study was approved by the Ethics Committee of the University of Medicine and Pharmacy of Craiova, Romania, and it was conducted in accordance with the Declaration of Helsinki and other international regulations in the field. Each patient included in our study signed an informed consent form so that data found in their medical records could be used for research purposes. No data could be used to identify a patient because each patient was assigned a specific code, and every piece of information that could lead to patient identification was deleted from the study database. The results of the study were reported in accordance with the Transparent Reporting of a Multi-Variable Prediction Model for the Individual Prognosis or Diagnosis statement [19,20].

### 2.1. Study Design and Patient Selection

**Figure 1:**
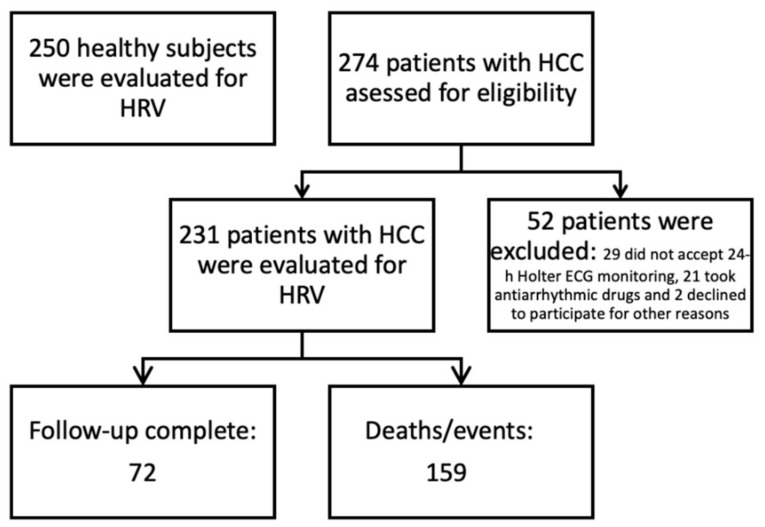
The design of the study. HCC: hepatocellular carcinoma; HRV: heart rate variability; ECG: electrocardiogram.

We retrospectively reviewed the medical records of 231 patients who were newly diagnosed with HCC between January 2014 and March 2018 at the Emergency County Hospital of Craiova (Romania), which is affiliated with the University of Medicine and Pharmacy of Craiova. These patients were monitored by Holter electrocardiogram (ECG) for 24 h before the initiation of any therapy. In accordance with the European Society for Medical Oncology (ESMO) clinical practice guidelines, a diagnosis of HCC is based on histological analysis and/or contrast-enhanced imaging findings [21]. In patients with liver cirrhosis and specific imaging criteria, the formal pathological proof is not mandatory for diagnosis, and the clinician can rely on the contrast-enhanced (CE) imaging criteria for the assessed lesion [21]. These criteria require a multi-phasic CECT (computed tomography) or CEMRI (magnetic resonance imaging). A diagnosis can be established if the typical vascular hallmarks of HCC are identified in a nodule of >1 cm in diameter using one of these two modalities. Serum alfa-fetoprotein (AFP) has no role in the diagnostic algorithm [21]. Patients who were <18 years of age, had an active infection or had received any medications that could affect HRV, such as beta-blockers, or other anti-arhythmic drugs, were excluded from our study. All patients included in this study had a sinus rhythm. Additionally, arrhythmias were part of the exclusion criteria of the study (both electrical stimulus production disorders and conduction disorders).To determine the cut-off values of HRV, 24 h Holter ECG recordings were analyzed for 250 healthy subjects while maintaining the proportions for age and gender groups with the patients included in the study. It should be noted that 274 newly diagnosed patients with HCC were initially evaluated for eligibility, but 52 of them were excluded: 29 did not accept 24-h Holter ECG monitoring, 21 took antiarrhythmic drugs, and 2 declined to participate for other reasons. Finally, 231 patients were enrolled in the study, of whom 72 survived at least until follow-up at 36 months, while 159 patients died within 36 months after receiving their diagnosis. The design of the study is shown in Figure 1.

### 2.2. Follow-up of the Patients

Serum AFP level and other laboratory tests were monitored upon patients’ inclusion in the study. The various demographic, medical history, serum biochemical, and clinical characteristics were analyzed at baseline. The clinical and pathological features assessed included age, gender, history of alcohol use, history of smoking, and hepatitis B or C. The following serum biochemical variables were analyzed: total bilirubin, serum albumin, alanine aminotransferase (ALT), aspartate aminotransferase (AST), serum creatinine, International Normalized Ratio (INR), AFP, white blood cell (WBC) count, absolute platelet count (PLT) and C-reactive protein (CRP). All patients were followed until death or until 28 February 2021.

### 2.3. Heart Rate Variability Assessment

Each patient included in our study was monitored by Holter ECG for 20–30 h. Patients who were subsequently diagnosed with HCC were eligible for the study, and those in whom the diagnosis was not confirmed were not included in the study. Holter ECG monitoring was performed using a TLC5000 Holter ECG (Contec Medical Systems, Qinhuangdao, Hebei Province, China) capable of performing an HRV analysis in both the time and frequency domains. The main indices analyzed in the time domain were the mean successive difference in normalized R–R intervals (MSD), the standard deviation of all normal-to-normal (NN) intervals (SDNN), the square root of the mean of the sum of the squares of differences between adjacent NN intervals (rMSSD), and the number of successive NN intervals differing by more than 50 ms divided by the total number of all NN intervals (pNN50) [22]. In the frequency domain, the following indices were analyzed: the ultra-low-frequency (ULF) band (≤0.003 Hz), the power of the very-low-frequency band (0.0033–0.04 Hz) of the HRV spectrum (VLF), the power of the low-frequency band (0.04–0.15 Hz) of the HRV spectrum (LF), and the power of the high-frequency band (0.15–0.4 Hz) of the HRV spectrum (HF) [22]. All HRV indices included in our study were analyzed for the entire monitored period, usually between 20 and 30 h, for each patient included in the study.

### 2.4. Assessment of Norepinephrine Transporter

To analyze nervous influences at the local level, we chose to evaluate the expression of the norepinephrine transporter. We included 48 patients with HCC who underwent surgery. The formalin-fixed and paraffin-embedded resection tissues of those patients were sectioned to 3 μm in thickness and de-paraffinized, then rehydrated and processed for antigen retrieval. The slides were further incubated with norepinephrine transporter monoclonal primary antibody (CL3063)/NBP2-62704 (dilution 1:20; Novus Biological, Abingdon, UK) at 4 °C for 18 h. Finally, the signal was found via 3, 3′-diaminobenzidines (DAB) (Dako, Glostrup, Denmark). Subsequently, after hematoxylin and eosin staining, the slides were cover-slipped in DPX (Sigma-Aldrich, St. Louis, MO, USA).

### 2.5. Statistical Analysis

All of the statistical analyses were performed using the latest version of SPSS software (IBM, Armonk, NY, USA) or, where appropriate, the latest version of GraphPad software (San Diego, CA, USA). The categorical data are reported as the number or percentage of observations, and continuous variables are reported as the mean and standard deviation. To compare the means of the two groups, we used the Student’s *t*-test. To compare the means of more than two groups, we used an ANOVA test. Univariate and multivariate analyses of the relationships between overall survival (OS) and the study variables were assessed using Cox proportional hazard models. Variables that were shown to be associated with OS in the univariate analysis were evaluated in the multivariate Cox proportional hazard model. A receiver operating characteristic (ROC) curve was designed to establish the cut-off value for each HRV study variable, and the area under the curve (AUC) was calculated to evaluate the discriminatory capacity of each. It should be noted that the cut-off value was calculated keeping a balance between sensitivity and specificity, and the patients were divided into two groups based on the HRV indices’ cut-off values. A Kaplan–Meier survival analysis was performed to compare the OS of the patients included in different groups, and the significance of the intergroup difference was evaluated using the log-rank test. A Pearson correlation analysis was performed to determine the relationship between HRV and clinical-pathological features. In all cases, *p* < 0.05 was considered statistically significant.

## 3. Results

### 3.1. Assessment of Cut-off Value

**Figure 2:**
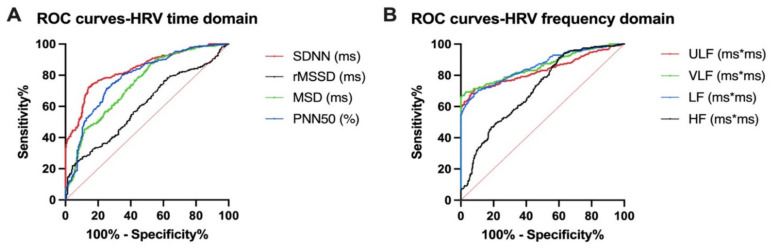
Receiver operating characteristic (ROC) curve analysis in the time domain (**A**) and in the frequency domain (**B**). MSD: mean successive difference in normalized R–R intervals; SDNN: standard deviation of all normal-to-normal (NN) intervals; rMSSD: square root of the mean of the sum of the squares of differences between adjacent NN intervals; pNN50: number of successive NN intervals differing more than 50 ms divided by the total number of all NN intervals. ULF: the ultra-low-frequency band; VLF: the power of the very-low-frequency band of the HRV spectrum; LF: the power of a low-frequency band of the HRV spectrum; HF: the power of a high-frequency band of the HRV spectrum.

**Figure 3:**
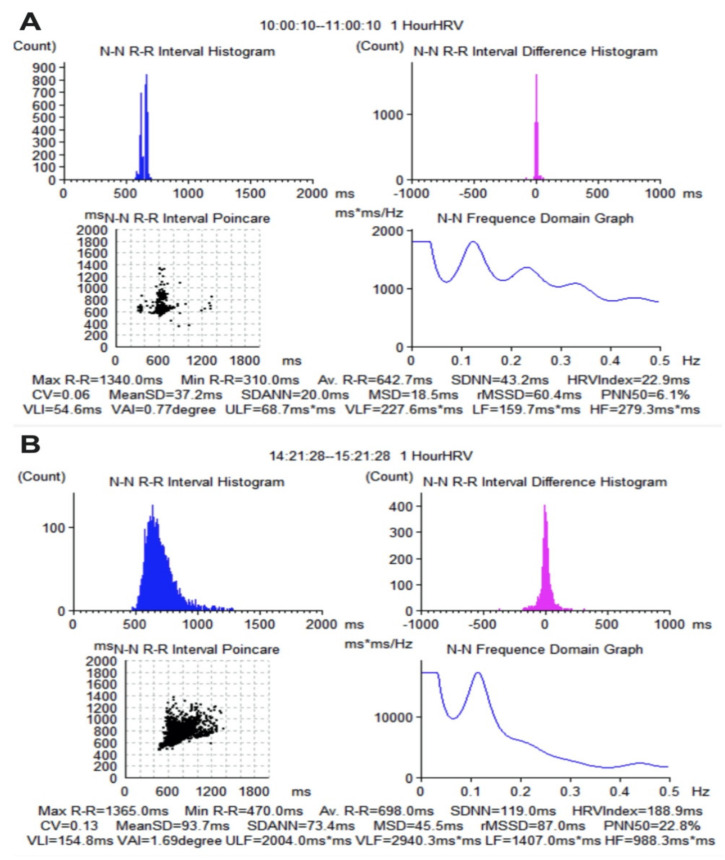
Representative images with HRV indices automatically determined by the ECG Holter software in a patient with HCC (**A**) and in a healthy subject (**B**).

We used an overall survival (OS) of 3 years as the primary endpoint. For each parameter in the HRV analysis, we determined a cut-off value, according to which the patients were included in two groups—a value that struck a balance between sensitivity and specificity. The optimal cut-off values for HRV indices in the time domain were SDNN 110 ms (AUC = 0.8404, SE = 0.01794, 95% CI = 0.8052 to 0.8756, *p* < 0.000), MSD 49.2 ms (AUC = 0.7436, SE = 0.02213, 95% CI = 0.7002 to 0.7870, *p* < 0.0001), rMSSD 91 ms (AUC = 0.6059, SE = 0.02561, 95% CI = 0.5557 to 0.6561, *p* < 0.0001), PNND50% 23.44 ms (AUC = 0.7819, SE = 0.02096, 95% CI = 0.7408 to 0.8229, *p* < 0.0001). In the frequency domain, we established the following cut-off values by means of ROC curves: ULF 860.3 (AUC = 0.8327, SE = 0.01979, 95% CI = 0.7939 to 0.8715, *p* < 0.0001), VLF 2438 (AUC = 0.8596, SE = 0.01763, 95% CI = 0.8251 to 0.8942, *p* < 0.0001), LF 911 (AUC = 0.8586, SE = 0.01721, 95% CI = 0.8249 to 0.8923, *p* < 0.0001), HF 805.2 (AUC = 0.7084, SE = 0.02324, 95% CI = 0.6629 to 0.7540, *p* < 0.0001). The ROC curves for HRV indices are shown in Figure 2. Representative images with HRV indices are shown in Figure 3.

### 3.2. Patients and Tumor Clinicopathological Features

Between January 2014 and March 2018, the patients included in our study were monitored by Holter ECG for 24 h from the time of diagnosis of hepatocellular carcinoma. Overall, 231 patients were followed up for up to 36 months post-recruitment. Of these, 148 (64.1%) were male, while 83 (35.9%) were female. We also observed that 106 (45.9%) patients were diagnosed with hepatitis B, while only 46 (19.9%) were diagnosed with hepatitis C. The mean tumor size (determined on imaging evaluation) was 7.73 cm (with an interval between 1.2 and 27 cm) at the greatest diameter, and 123 (53.2%) patients had tumors ≥ 5 cm in diameter. Increased AFP levels (> 400 ng/mL) were observed in 51 patients (22.1%). We highlight all the variables evaluated at baseline in Table 1, depending on the SDNN 110 ms cut-off value.

### 3.3. Risk Factors for Poor Overall Survival

Univariate analysis demonstrated that age > 60 years, hepatitis C, portal vein involvement (thrombosis), tumor size > 5 cm, AFP > 400 ng/mL, serum albumin, and CRP were risk factors for poor OS (Table 2). Gender, hepatitis B, a history of alcohol use, a history of smoking, serum ALT, serum AST, total bilirubin, INR, creatinine, platelets, and white blood cells were not significantly related to OS. The variables that showed a statistically significant difference in the univariate analysis were introduced in the multivariate analysis, and we found that only AFP > 400 ng/mL and tumor size > 5 cm were independent risk factors for poor OS. Regarding HRV indices, the univariate analysis indicated that SDNN < 110 ms, MSD < 49.2 ms, PNN50 < 23.4%, ULF < 860.3 ms * ms, VLF < 2438 ms * ms, LF < 911.2 ms * ms, and HF < 805.2 ms * ms were risk factors for poor OS, while rMSSD < 91 ms was not significantly related to OS. The same statistically significant differences were found in the multivariate analysis, with the exception of MSD < 49.2ms and rMSSD < 91 ms (Table 2). It should be mentioned that in both the univariate analysis and in the multivariate analysis, among the HRV SDNN indices, <110 ms was an independent risk factor for OS with an HR of 3.646 (95% CI 2.143 to 6.05).

### 3.4. Association between HRV Indices and Survival

**Figure 4:**
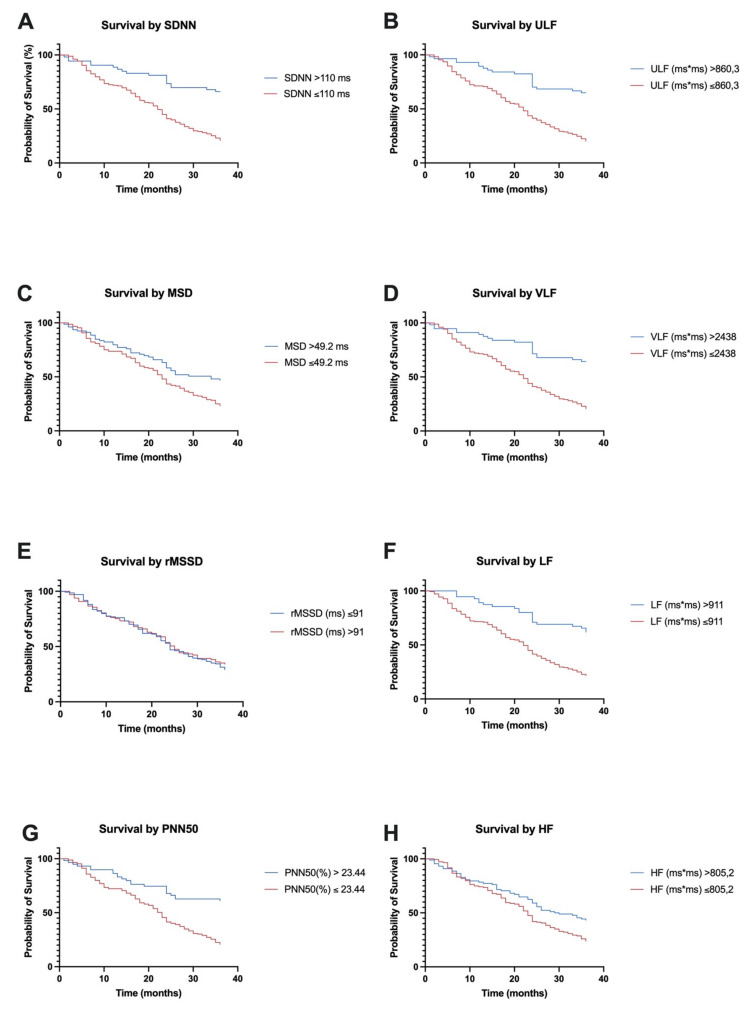
Kaplan–Meier survival curves according to HRV indices. (**A**,**C**,**E**,**G**): survival by time-domain indices. (**B**,**D**,**F**,**H**): survival by frequency domain indices.

At 3 years of enrollment in the study, the survival rate for patients was 31.16%, with 72 patients surviving a full follow-up of 36 months. Patients with SDNN > 110 ms had a 36-month survival rate of 66.03% compared to SDNN < 110 ms, where the survival rate was only 20.78% (HR = 3.432, 95% CI of ratio 2.448 to 4.811, *p* < 0.000). High survival rates were also recorded in patients with MSD > 49.2 ms (46.83% vs. 23.02% at the 3-year follow-up, HR = 1.731, 95% CI of ratio 1.257 to 2.383, *p* = 0.010) and PNN50 > 23.44 ms (61.01% vs. 20.71% at the 3-year follow-up, HR = 2.789, 95% CI of ratio = 1.997 to 3.896, *p* < 0.000) as opposed to rMSSD, where no different survival rates were recorded at the cut-off value of 91 ms (29.10% vs. 34.02%, HR = 1.093, 95% CI of ratio = 0.7938 to 1.504, *p* = 0.5865). In the frequency domain, for all four analyzed indices, high survival rates were recorded in people with values above the cut-off value, as follows: for ULF > 64.91% vs. 20.11% (HR = 3.412, 95% CI of ratio = 2.451 to 4.750, *p* < 0.000), for VLF > 64.28% vs. 20.57% (HR = 3.282, 95% CI of ratio = 2.352 to 4.579, *p* < 0.000), for LF > 61.81% vs. 21.59% (HR = 3.096, 95% CI of ratio = 2.219 to 4.320, *p* < 0.000) and for HF > 43.18% vs. 23.77% (HR = 1.572, 95% CI of ratio = 1.147 to 2.155, *p* = 0.027). All of these data are shown in Figure 4. We also performed an analysis of the HRV indices by combining them. We observed that patients who presented with a decrease in all HRV indices in the time domain (SDNN < 110 ms, MSD < 49.2 ms, rMSSD < 91 ms, and PNND50% < 23.44 ms) were associated with a 3-year follow-up survival of only 18.5% compared to patients who did not have low values for all of these indices (45.79%, *p* = 0.006, HR = 1.565, 95% CI of ratio = 1.136 to 2.156). Additionally, patients who presented with a decrease in all HRV indices in the frequency range (ULF <860.3, VLF < 2438, LF < 911, and HF < 805.2) had a 3-year follow-up survival of 23.42%, compared to patients who did not have low values for all of these indices (38.33%, *p* = 0.000, HR = 1.715, 95% CI of ratio = 1.248 to 2.358).

### 3.5. Assessment of Norepinephrine Transporter

**Figure 5:**
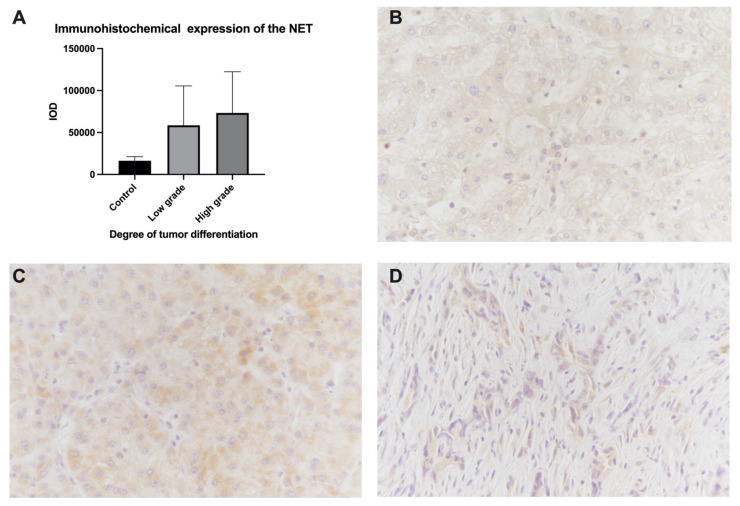
(**A**): Immunohistochemical expression of the norepinephrine transporter (IOD). Examples of microscopic images with normal liver tissue, (**B**) low-grade (**C**), and high-grade (**D**) tumor tissue. NET expression is observed in brown. Magnification: 400×. IOD: integrated optical density; NET: norepinephrine transporter.

Since we observed that in patients with HCC, low values of HRV indices predominate, we concluded that they are caused by the increased activity of the sympathetic nervous system. Thus, we attempted to ascertain whether we could identify this at the tumor level by analyzing the immunohistochemical expression of the norepinephrine transporter (NET). The control tissue was obtained by analyzing the margins of tumor resection. For the analysis of the immunohistochemical expression of NET, we used integrated optical density (IOD). Thus, we found an average IOD for NET in the control tissue of 16,289 ± 8518; in the tissue with low-grade tumor tissue, the average was 58,506 ± 141,177; and, in the tissue with high-grade tumor tissue, the average was 73,262 ± 139018. We found a higher mean NET in the tumor tissue, but due to the very high variability of NET expression, no statistically significant differences were noted (Figure 5).

## 4. Discussion

The present study described 24 h HRV indices using the time domain and frequency domain in patients with HCC at the time of diagnosis, as previously described [10,22,23]. We found that low HRV indices correlate with low survival rates. The first study that looked at the influences of the autonomic nervous system via HRV and survival in HCC was conducted 10 years ago. That study was prospective and concluded that there was a link between low survival rates and HRV indices [23]. The main limitation of the study was the short duration of the follow-up period (3 months), and the small number of patients included. Compared to this, the duration of the follow-up period in our study was 3 years. Among the other notable aspects of our study are the cut-off values of HRV indices, which were determined by comparison with healthy subjects.

Several studies have been performed on the correlation between HRV indices and the prognoses of cancer patients. Giese-Davis et al. demonstrated in 2015 that vagal activity, objectified by elevated HF-HRV values, would predict a longer survival time in patients with metastatic or recurrent breast cancer [24]. Another study showed that a cut-off value of SDNN < 70 ms is associated with a shorter survival time in patients with different cancers [25]. It should be noted that, in our study, the cut-off value of SDNN was 110 ms, but this was determined by comparing the group of HCC patients with healthy subjects. An SDNN value < 121 ms, alongside other low parameters in the HRV time-domain (rMSSD, PNN50, and SDANN), was found in patients with acute leukemia [26]. As such, there is no consensus on the cut-off values for HRV indices. These depend very much on the duration of HRV analysis. On the other hand, a recent study published by Strous MTA et al. highlighted the fact that HRV was found to have no prognostic value in patients with primary colorectal cancer who underwent curative surgical treatment because low HRV indices were not associated with reduced overall survival [27]. Moreover, low HRV was not significantly associated with elevated CEA levels during follow-up or postoperative complications [27]. The duration of HRV analysis was only 10 s because, in that study, only standard electrocardiograms were used for HRV analysis, which records cardiac electrical activity for only 10 s. In comparison, the duration of HRV analysis in our study was between 20 and 30 h. Other cancers in which the prognostic value of HRV reduction was analyzed were pancreatic cancer [17], prostate and non-small cell lung cancer [28], and gastric cancer [29].

The main hypothesis that explains why reduced HRV is associated with a negative prognosis in cancers is represented by the fact that, at the molecular level, the sympathetic nervous system predominates to the detriment of parasympathetic nervous activity [18]. A study on cell cultures showed that hepatocellular carcinoma cells expressed adrenergic receptors [30]. This observation supports the results of our analysis, especially since, at the molecular level, it is well known that epinephrine and norepinephrine have the ability to increase the migratory capacity of cancer cells [31]. Taking these observations into account, we analyzed the immunohistochemical expression of the norepinephrine transporter in liver tumor tissue and observed an increase in tumor tissue compared to normal liver tissue, without being able to show a statistically significant difference. Moreover, a meta-analysis of 23 clinical trials that included over 2600 patients with cirrhosis indicated that the use of non-selective sympathetic beta-blockers (such as propranolol) reduces the risk of these patients developing hepatocellular carcinoma [32]. At the opposite pole is the activity of the parasympathetic nervous system, which, according to HRV indices, is reduced in HCC patients. This reduction contributes to increased oxidative stress and excessive inflammation [33,34]. Last but not least, it should be noted that the reduction in vagal activity may be due to the use of chemotherapeutics in the treatment of cancer [35].

## 5. Conclusions

HRV measurement is an easy and safe method to assess autonomic dysfunction. This study demonstrates that HRV indices identify HCC patients at high risk of death and suggests that such monitoring might guide the need for early therapy in such patients, as well as the fact that HRV can potentially be a noninvasive biomarker for HCC prognosis. More large prospective multicenter randomized controlled trials are needed to validate these observations.

## Figures and Tables

**Table 1:** Baseline clinical–pathological characteristics of hepatocellular carcinoma patients, stratified by SDNN 110 ms cut-off value.

		SDNN < 110 ms	SDNN > 110 ms	
Variable	Category	Value	Value	*p*-Value
Age (years)	<60	105 (45.5%)	50 (21.6%)	0.001*
	>60	73 (31.6%)	3 (1.3%%)	
Gender	Female	59 (25.5%)	24 (10.4%)	0.106
	Male	119 (51.5%)	29 (12.6%)	
Hepatitis B	Yes	85 (36.8%)	21 (9.1%)	0.297
	No	93 (40.3%)	32 (13.9%)	
Hepatitis C	Yes	36 (15.6%)	10 (4.3%)	0.828
	No	142 (61.5%)	43 (18.6%)	
History of alcohol use	Yes	36 (15.6%)	9 (3.9%)	0.601
	No	142 (61.5%)	44 (19%)	
History of smoking	Yes	54 (23.4%)	13 (5.6%)	0.413
	No	124 (53.7%)	40 (17.3%)	
Portal vein involvement	Yes	49 (21.2%)	13 (5.6%)	0.665
	No	129 (55.8%)	40 (17.3%)	
Tumor size (>5 cm)	Yes	102 (44.2%)	21 (9.1%)	0.024 *
	No	76 (32.9%)	32 (13.9%)	
AFP (>400 ng/mL)	Yes	42 (18.2%)	9 (3.9%)	0.302
	No	136 (58.9%)	44 (19.0%)	
ALT (U/L)		77.3 ± 24.3	73.9 ± 31.3	0.410
AST (U/L)		88.6 ± 24.8	84.2 ± 32.2	0.287
Bilirubin (mg/dL)		0.96 ± 0.28	0.98 ± 0.29	0.735
INR		1.31 ± 0.85	1.26 ± 0.21	0.684
Albumin (g/dL)		3.95 ± 0.38	4.04 ± 0.44	0.202
Creatinine (mg/dL)		0.92 ± 0.20	0.91 ± 0.20	0.802
Platelets/mm^3^		162,608.3 ± 35,398.6	166,131.2 ± 39,530.1	0.536
WBC (×10^3^)/mm^3^		8.17 ± 2.69	8.44 ± 2.58	0.519
CRP (mg/L)		6.55 ± 2.57	6.26 ± 2.52	0.476

AFP: alfa-fetoprotein; ALT: alanine aminotransferase; AST: aspartate aminotransferase; INR: International Normalized Ratio; WBC: white blood cell count; PLT: absolute platelet count; CRP: C-reactive protein; SDNN: standard deviation of all normal-to-normal (NN) intervals; * *p* < 0.05.

**Table 2:** Univariate and multivariate assessment to identify predictors of overall survival in hepatocellular carcinoma patients.

Variable	Hazard Ratio (95% CI)	*p*-Value	Hazard Ratio (95% CI)	*p* Value
Gender (male)	1.240 (0.890–1.728)	0.204		
Age (>60 years)	1.615 (1.172–2.224)	0.003 ^#^	1.159 (0.833–1.613)	0.382
Hepatitis B (yes)	1.084 (.7941.481)	0.610		
Hepatitis C (yes)	1.457 (1.004–2.115)	0.047 ^#^	1.189 (0.810–1.747)	0.377
History of alcohol use (yes)	1.220 (0.832–1.790)	0.309		
History of smoking (yes)	1.289 (0.920–1.805)	0.139		
Portal vein involvement (yes)	1.422 (1.013–1.996)	0.042 ^#^	1.310 (0.930–1.845)	0.123
Tumor size (>5 cm)	2.367 (1.708–3.279)	0.000 ^#^	2.117 (1.509–2.972)	0.000 ^#^
AFP (>400 ng/mL)	1.655 (1.159–2.364)	0.006 ^#^	1.658 (1.141–2.409)	0.008 ^#^
ALT (U/L)	1.004 (0.999–1.010)	0.132		
AST (U/L)	1.005 (1.000–1.011)	0.071		
Bilirubin (mg/dL)	0.931 (0.532–1.629)	0.802		
INR	1.077 (0.930–1.247)	0.321		
Albumin (g/dL)	0.663 (0.456–0.964)	0.031 ^#^	1.008 (0.676–1.504)	0.967
Creatinine (mg/dL)	1.663 (0.782–3.539)	0.186		
Platelets/mm^3^	0.946 (0.835–1.233)	0.141		
WBC (x10^3^)/mm^3^	1.021 (0.962–1.083)	0.503		
CRP (mg/L)	1.378 (0.946–2.009)	0.023 ^#^	1.319 (1.237–1.406)	0.075
SDNN < 110 ms	3.501 (2.138–5.732)	0.000 ^#^	3.646 (2.143–6.205)	0.000 ^#^
MSD < 49.2 ms	1.693 (1.192–2.404)	0.003 ^#^	1.378 (0.946–2.009)	0.095
rMSSD < 91 ms	1.082 (0.789–1.484)	0.626		
PNN50 < 23.4%	2.790 (1.816–4.287)	0.000 ^#^	2.430 (1.523–3.877)	0.000 ^#^
ULF < 860.3 ms * ms	3.479 (2.172–5.574)	0.000 ^#^	3.436 (2.056–5.745)	0.000 ^#^
VLF < 2438 ms * ms	3.229 (2.016–5.172)	0.000 ^#^	3.227 (1.940–5.368)	0.000 ^#^
LF < 911.2 ms * ms	3.147 (1.984–4.992)	0.000 ^#^	2.832 (1.754–4.572)	0.000 ^#^
HF < 805.2 ms * ms	1.577 (1.127–2.206)	0.008 ^#^	1.441 (1.011–2.054)	0.043 ^#^

CI: confidence interval; AFP: alfa-fetoprotein; ALT: alanine aminotransferase; AST: aspartate aminotransferase; INR: International Normalized Ratio; WBC: white blood cell count; PLT: absolute platelet count; CRP: C-reactive protein; MSD: mean successive difference in normalized R–R intervals; SDNN: standard deviation of all normal-to-normal (NN) intervals; rMSSD: square root of the mean of the sum of the squares of differences between adjacent NN intervals; pNN50: number of successive NN intervals differing more than 50 ms divided by the total number of all NN intervals. ULF: the ultra-low-frequency band; VLF: the power of the very-low-frequency band of the HRV spectrum; LF: the power of a low-frequency band of HRV spectrum; HF: the power of a high-frequency band of HRV spectrum; ^#^ *p* < 0.05.

## Data Availability

The data presented in this study are available on request from the corresponding authors.
